# Editorial: Mechanisms and management of inflammation-driven cardiovascular risk—from obesity and diabetes to autoimmunity and cancer

**DOI:** 10.3389/fcvm.2026.1823320

**Published:** 2026-03-19

**Authors:** Thiago Quinaglia, Otavio Coelho-Filho, Andrei C. Sposito

**Affiliations:** Discipline of Cardiology, Department of Medicine, Faculty of Medical Science, State University of Campinas, Campinas, Brazil

**Keywords:** athero-thrombotic disease, cardiovascular risk, chronic kidney disease, HIV, inflammation, oncology, psoriasis, rheumatoid anhritis

Commonly viewed as a risk “modifier”, inflammation should more accurately be seen as a core driver of cardiovascular risk that links metabolic disease, chronic infection, autoimmunity, cancer, and treatment-related toxicities to atherothrombosis, myocardial injury, and adverse remodeling. In this Research Topic, 13 articles collectively reinforce a unifying message: inflammation shapes both the substrate and the triggers of cardiovascular events, and its clinical consequences extend beyond classic atherosclerotic cardiovascular disease (ASCVD) to include thrombotic complications in cancer, fibrotic myocardial remodeling in chronic inflammatory states, and cardiotoxicity signals in immune-oncology. At the same time, the Research Topic highlights a practical challenge—how to measure inflammation-driven risk with sufficient precision to guide prevention and management across heterogeneous patient populations.

A central theme emerging across the papers is the concept of residual inflammatory risk. Even when low-density lipoprotein cholesterol is treated and guideline-directed therapy is in place, many patients remain exposed to ongoing systemic inflammation that sustains plaque activity, perturbs endothelial function, and amplifies thrombogenicity (1). In a nationwide primary care analysis from Spain, Pesce et al. report that systemic inflammation (CRP ≥ 2 mg/L) is present in a majority of patients with ASCVD and is even more prevalent among those with comorbid chronic kidney disease (CKD). This epidemiologic observation is clinically consequential for two reasons. First, it aligns with the concept that CKD is a prototypical state of “inflammation plus toxin accumulation,” in which immune activation, oxidative stress, and metabolic derangements converge to promote vascular disease. Second, it underscores a persistent treatment gap: patients with systemic inflammation in this cohort were less frequently dispensed statins and aspirin than those without inflammation, raising the possibility that those at highest inflammatory risk may also be less optimally protected. Inflammatory burden is therefore not only a biological phenomenon but also a marker for clinical complexity and undertreatment.

The mechanistic bridge from inflammation to cardiovascular events is frequently thrombotic. Inflammation promotes thrombosis through multiple, mutually reinforcing pathways: endothelial activation, increased tissue factor expression, platelet priming, neutrophil extracellular traps (NETs), and dysregulation of fibrinolysis (2). This Research Topic emphasizes that inflammation-driven thrombosis is particularly prominent in oncology, where malignancy and vascular devices provide potent prothrombotic contexts. Wang et al. address the issue of central venous catheter-related thrombosis (CRT), a common and clinically important complication in cancer care, by developing and validating a nomogram in a large cohort of 4,691 patients. Notably, 70% of CRT occurred within the first week after catheter insertion, emphasizing that thrombotic risk is not only patient-related but also procedure- and time-dependent. The strength of this work lies less in identifying a single “culprit biomarker” and more in providing a pragmatic, multivariable risk tool that can support early preventive strategies. Inflammation is not always explicitly quantified in CRT prediction, yet the clinical phenotype of cancer-associated thrombosis is deeply intertwined with inflammatory signaling, tumor–host interactions, and treatment effects. Tools such as this nomogram may thus serve as operational proxies for inflammation-thrombosis biology, enabling clinicians to identify those who require intensified surveillance or prophylaxis during the highest-risk window.

Cancer risk itself is also shown to be intimately related to systemic inflammation, particularly in the context of obesity and metabolic dysfunction (3). In a large prospective cohort of 356,554 participants with 9,048 incident cancers, Bian et al. demonstrate that multiple inflammation-related blood biomarkers correlate with overall cancer incidence and partially mediate the association between anthropometric measures and cancer risk. Higher CRP and globulin levels were associated with higher cancer risk, and composite inflammatory indicators showed robust associations—stronger for obesity-related cancers than for non–obesity-related cancers. These findings complement the overarching theme of the collection unveiling that the metabolic–inflammator*y* axis is not simply a cardiovascular risk amplifier, but also a biological hallway connecting adiposity to carcinogenesis. Importantly, the same inflammatory pathways implicated in tumor initiation and progression, also contribute to vascular dysfunction and thrombosis. Thus, obesity and diabetes, through chronic low-grade inflammation, may generate a “double burden” risk of cancer susceptibility and cardiovascular event propensity.

In this context, biomarkers that integrate metabolic and inflammatory information are increasingly attractive for risk stratification. Song et al. review the triglyceride glucose–body mass index (TyG-BMI) index, positioning it as a practical surrogate for insulin resistance that correlates with multisystem diseases, including cardiovascular disease, diabetes, fatty liver disease, hyperuricemia, and cancer. While TyG-BMI is not an inflammatory biomarker *per se*, insulin resistance is tightly linked to inflammation via adipose tissue macrophage activation, altered adipokines, and endothelial dysfunction. In this collection, TyG-BMI points toward integrated risk markers that are feasible at scale and may capture upstream inflammation drivers of cardiovascular risk. Complementing this metabolic framing, Huang et al. use NHANES data to show that better cardiovascular health, measured by Life's Essential 8, is associated with lower systemic immune-inflammatory burden as indexed by the Systemic Immune-Inflammation Index (SII). Their findings reinforce a clinically actionable point: improving lifestyle and health factors is an anti-inflammatory intervention, with measurable effects. Together, these studies suggest that a future stratification paradigm may blend classic risk factors, metabolic indices (e.g., TyG-BMI), and inflammatory measures (e.g., CRP, SII) to identify patients whose risk is disproportionately driven by inflammation.

Yet the translation of inflammation biology into cardiovascular prediction and management remains uneven. Silva et al., in a scoping review summarize a striking limitation of traditional ASCVD scores. These tools often underperform in inflammatory-driven conditions and may misclassify a substantial portion of individuals, particularly those in intermediate-risk categories where clinical decisions are most sensitive. The review further emphasizes that in secondary prevention, a sizable fraction of patients exhibit persistently elevated high-sensitivity C-reactive protein (CRP), consistent with residual inflammatory risk that correlates with adverse outcomes. This literature mapping is essential because it frames the editorial objective of this Research Topic. More than enumerating inflammatory associations, arguing for structured incorporation of inflammation into clinical decision-making, particularly in populations where inflammation is a primary driver (e.g., autoimmune disease, CKD, chronic infections, cancer).

Autoimmune disease provides a compelling test case for inflammation-aware stratification. Che et al. focus on rheumatoid arthritis (RA), a condition characterized by systemic inflammation and immune dysregulation, and demonstrate that coronary heart disease (CHD) risk in RA is shaped by both traditional and disease-related factors. Their nomogram identifies hypertension and HbA1c alongside RA duration, carotid plaque burden, uric acid, and ECG abnormalities as independent predictors of CHD in RA, with strong discrimination (AUC ∼0.87). These results reinforce that inflammation-driven cardiovascular risk rarely operates in isolation. It rather interacts with metabolic factors (e.g., glycemic control), vascular injury markers (carotid plaque), and cumulative inflammatory exposure (disease duration) (4). Such models can help clinicians move from generalized statements (“RA increases CHD risk”) to individualized estimation that supports targeted prevention. In parallel, Long et al. address psoriasis, using Mendelian randomization to test causality for cancer risk. Their negative findings (no evidence of a causal genetic link between psoriasis and overall or site-specific cancers) remind us that associations observed in inflammatory diseases may reflect shared environmental exposures, treatment effects, surveillance bias, or non-genetic inflammatory pathways, rather than direct genetic causation. This methodological contrast is important for the Research Topic. Inflammation may still be mechanistically important even when Mendelian randomization does not support a direct genetic causal pathway, because inflammation is shaped by dynamic immune–environmental interactions not fully captured by genetic instruments.

Chronic infection and immune activation also appear prominently in this collection. Hope Mutengo et al. synthesize cardiac magnetic resonance (CMR) observational evidence linking HIV infection to myocardial fibrosis, reporting higher prevalence of late gadolinium enhancement and increased native T1 and extracellular volume fraction in people with HIV compared to uninfected controls. Fibrosis can be interpreted as a cumulative footprint of chronic inflammation, immune activation, microvascular dysfunction, and potentially cardiotoxic exposures. Myocardial fibrosis predisposes to heart failure, arrhythmia, and adverse outcomes (5). From the perspective of inflammation-driven cardiovascular risk, the HIV–fibrosis association reveals that inflammatory injury can manifest not only as accelerated atherosclerosis but also as structural myocardial remodeling, broadening the clinical endpoints that inflammation-aware management should consider.

The complexity between inflammation and cardiovascular disease is further enhanced by modern immunomodulatory therapies. Immune checkpoint inhibitors (ICI) exemplify a therapeutic paradigm in which enhancing immune activity can yield dramatic oncologic benefit while occasionally precipitating severe immune-mediated toxicity, including myocarditis (6). Celebi Coskun et al. evaluate whether left ventricular global longitudinal strain (LV GLS) detects early ICI-related cardiac dysfunction; in their retrospective cohort, LV GLS did not demonstrate a clear predictive role, though a small number of adverse events occurred. Even if definitive conclusions are limited by sample size and event scarcity, this study highlights a critical need. From a mechanistic standpoint, ICI myocarditis is an archetypal example of inflammation-driven myocardial injury with potentially thrombotic and arrhythmic sequelae. Clinically, it reinforces that as immunotherapies expand, cardiovascular care must adapt to recognize immune-inflammatory toxicities early, integrate imaging and biomarkers judiciously, and balance false positives against the catastrophic nature of missed myocarditis.

Inflammation also drives risk through pathways that are not always captured by conventional inflammatory biomarkers. CKD illustrates this through the accumulation of uremic toxins that disrupt cardiovascular biology. Harlacher et al. identify mycophenolic acid-*β*-glucuronide (MPA-G) as a metabolite accumulating in patients with kidney failure that adversely affects cardiomyocyte metabolic activity, increases mitochondrial reactive oxygen species, and impairs respiration *in vitro* at clinically relevant concentrations. This work is notable because it adds granularity to a common clinical situation, namely, immunosuppression with mycophenolate mofetil in patients with renal impairment. It suggests that beyond inflammation, toxin–mitochondria interactions may constitute an underappreciated mechanism contributing to cardiovascular vulnerability in CKD populations, which often already exhibit systemic inflammation and high thrombotic risk. In the context of the Spanish database study (Pesce et al.) showing high systemic inflammation prevalence among ASCVD patients with CKD, these findings collectively argue for a multidimensional CKD cardiovascular risk model that includes inflammatory status, uremic toxin profiles, and medication metabolism considerations.

From acute coronary syndromes to chronic CAD, this Research Topic also returns to classic cardiovascular endpoints, reframed through inflammatory signaling and biomarker discovery. Guan et al. evaluate arterial blood expression of low-density lipoprotein receptor-related protein 1 (LRP1) in ST-segment elevation myocardial infarction (STEMI) patients undergoing percutaneous coronary intervention (PCI) and report that LRP1 expression is lower in STEMI than controls, yet higher LRP1 tertiles are associated with increased six-month MACE and correlate with injury and dysfunction markers (NT-proBNP, cTnT, and lower LV ejection fraction). These data suggest LRP1 may capture aspects of post-infarction pathobiology related to inflammation, remodeling, and recovery. The apparent paradox of lower LRP1 in STEMI overall but higher LRP1 predicting worse outcomes within STEMI patients invites mechanistic exploration. LRP1 is implicated in lipid metabolism, inflammation modulation, and cellular signaling (7). Clinically, this study contributes to the theme of refining post-PCI prognostication with biomarkers that may reflect ongoing inflammatory remodeling and vulnerability to recurrent events.

Finally, the collection includes an innovative attempt to repurpose anti-proliferative cancer therapeutics for vascular inflammation in chronic CAD. Marinho et al. test the effect on plaque progression of paclitaxel delivered via LDL-like nanoparticles in a double-blind pilot trial among patients with multivessel CAD on optimized medical therapy. They report favorable safety and a non-significant trend toward reduced IL-6 and hsCRP without clear differences in plaque progression on coronary computed tomography angiography (CCTA). Even as a pilot, the work is conceptually aligned with inflammation-driven risk management. It explores whether targeted delivery can reduce toxicity and potentially modulate vascular inflammation. In a field shaped by proof-of-concept anti-inflammatory trials, approaches that achieve inflammation reduction without unacceptable adverse effects remain of high interest. The negative or neutral imaging outcome also serves as a reminder that biomarker changes do not always translate into short-term structural plaque changes, particularly in small studies with heterogeneous plaque phenotypes and background therapies.

Taken together, the 13 articles form an integrated narrative across the Research Topic's theme. Inflammation is the common currency linking obesity and insulin resistance, diabetes, autoimmune disease, chronic infection, kidney failure, and cancer biology and treatment to cardiovascular outcomes. The relationship between inflammation and thrombotic risk appears in multiple aspects—device-related thrombosis in cancer, systemic inflammatory states in ASCVD and CKD, and the broader conceptual framework that inflammation destabilizes plaques and primes the coagulation system. Beyond thrombosis, inflammation influences disease occurrence and progression through carcinogenesis pathways, myocardial fibrosis, and therapy-induced immune-mediated injury. Critically, the collection emphasizes that management must evolve from recognizing inflammation as a risk factor to operationalizing it through risk models, biomarker-informed stratification, and interventions that address both metabolic drivers and immune activation.

Inflammation-driven cardiovascular risk should be conceptualized as multidomain: (i) systemic markers (CRP, immune-inflammatory indices), (ii) metabolic surrogates (TyG-BMI, HbA1c), (iii) disease-specific burden (RA duration, HIV status, CKD stage), (iv) structural and functional cardiac imaging (fibrosis on CMR, LV strain), and (v) thrombosis triggers (catheters, malignancy, acute coronary syndromes). Second, risk stratification should be designed to identify the patients in whom inflammation is not merely present but actionable—those likely to benefit from intensified prevention, closer monitoring, or targeted anti-inflammatory strategies. Third, the Research Topic highlights that future studies should integrate inflammation measures into prospective risk tools, evaluate incremental predictive value beyond traditional factors, and test whether reducing inflammatory burden results in meaningful reductions in thrombotic and cardiovascular endpoints across inflammatory-driven conditions.

In an era where cardiometabolic disease, immune-mediated disorders, CKD, and cancer increasingly coexist, the shared mechanistic denominator of inflammation offers both a challenge and an opportunity. The challenge is heterogeneity, including different triggers, immune pathways, and endpoints across diseases. The opportunity is unification. A coherent framework in which inflammation-driven cardiovascular risk is recognized, measured, and managed as a central pillar of precision prevention and cross-disciplinary care.
